# Copper Pollution Increases the Relative Importance of Predation Risk in an Aquatic Food Web

**DOI:** 10.1371/journal.pone.0133329

**Published:** 2015-07-14

**Authors:** Christopher Kent Kwan, Eric Sanford, Jeremy Long

**Affiliations:** 1 Biology Department and Coastal & Marine Institute Laboratory, San Diego State University, San Diego, California, United States of America; 2 Bodega Marine Laboratory and Department of Evolution and Ecology, University of California Davis, Bodega Bay, California, United States of America; Institut Pluridisciplinaire Hubert Curien, FRANCE

## Abstract

Although the cascading impact of predators depends critically on the relative role of lethal predation and predation risk, we lack an understanding of how human-caused stressors may shift this balance. Emergent evidence suggests that pollution may increase the importance of predator consumptive effects by weakening the effects of fear perceived by prey. However, this oversimplification ignores the possibility that pollution may also alter predator consumptive effects. In particular, contaminants may impair the consumptive effects of predators by altering density-dependent interactions among prey conspecifics. No study has directly compared predator consumptive and non-consumptive effects in polluted versus non-polluted settings. We addressed this issue by using laboratory mesocosms to examine the impact of sublethal doses of copper on tri-trophic interactions among estuarine predator crabs *Cancer productus*, carnivorous whelk prey *Urosalpinx cinerea*, and the basal resource barnacles *Balanus glandula*. We investigated crab consumptive effects (whelks culled without crab chemical cues), non-consumptive effects (whelks not culled with crab chemical cues), and total effects (whelks culled with crab chemical cues) on whelks in copper polluted and non-polluted waters. Realistic copper concentrations suppressed the effects of simulated crab lethal predation (whelk culling) by removing density-dependent feeding by whelks. Specifically, reductions in conspecific density occurring in elevated copper levels did not trigger the normal increase in whelk consumption rates of barnacles. Weakened effects of fear were only observed at extremely high copper levels, suggesting consumptive effects were more sensitive to pollution. Thus, pollution may shape communities by altering the roles of predators and interactions among prey.

## Introduction

Predators strongly impact community structure and function through consumptive (eating) and non-consumptive effects (scaring) on prey [1–4]. The balance of these effects can influence food web dynamics and biogeochemical cycling [1,5,6]. When consumptive effects dominate, the energy and nutrients from prey are transferred to higher trophic levels. However, this transfer is impaired as non-consumptive effects become increasingly important. Although anthropogenic stressors such as climate change [7], overfishing [8], invasive species [9], and pollution [10] can influence predator-prey interactions, stressor impacts on the balance of these effects is poorly known.

Emerging evidence indicates that chemical pollution may weaken the non-consumptive effects of predators by disrupting antipredator behaviors of prey [11–13]. This suggests that predators may influence their communities largely through consumptive effects under polluted scenarios. However, this hypothesis remains difficult to evaluate because no study has tested the relative importance of predator consumptive and non-consumptive effects—along with resulting indirect effects—under polluted scenarios [14,15]. Direct tests are needed because pollutants may also reduce predator consumptive effects [16–18] either by altering predation rates or by altering conspecific interactions amongst prey.

We hypothesize that pollutants may interfere with density-dependent interactions and conspecific signaling in prey. Animals often change foraging rates in the presence of conspecifics because of changes in competitive and aggregative interactions [19,20] or because of reduced vigilance and predation risk [21,22]. Importantly, in some marine invertebrates, foraging increases with decreasing conspecific density [5,23], in contrast to many birds and mammals [24–26]. Because pollution may disrupt signaling among organisms [11–13,27], density-dependent changes in foraging may disappear in the face of chemical pollution. Ultimately, such changes could provide an additional mechanism by which pollution alters the consumptive effects of predators.

Copper pollution, especially from anti-fouling substances and runoff, commonly impacts coastal ecosystems near urban areas [28,29]. Copper impairs neural processes [30], protein function [31], and chemosensory abilities [12,13,27] across many taxa [32,33]. However, tests of the impact of copper beyond the organism-level are largely lacking [14,34]. This is surprising given that 1) copper commonly affects aquatic animal chemoreception [11–13,27] and 2) chemosensory abilities often underlie key interactions including finding prey, avoiding predators, and detecting conspecifics [35]. Thus, copper pollution may often shape key interactions between predators and prey. For example, copper increased the vulnerability of salmon by interfering with their ability to detect predators chemically [12,13]. Furthermore, conspecific interactions among prey, including those mediated by contact or by waterborne-chemical cues, may be influenced by copper pollution. For example, copper may impair the ability of prey to detect and respond to cues that provide information about conspecific density.

We performed three laboratory mesocosm experiments to examine the influence of copper pollution on the cascading effects of predators in a model estuarine food chain of crab predators (*Cancer productus*), whelk prey (*Urosalpinx cinerea*), and basal barnacle resources (*Balanus glandula*). We selected this food chain because 1) *C*. *productus* interacts with *U*. *cinerea* [9], 2) whelks can change feeding on barnacle prey when exposed to predation risk [4–5], 3) there is an established protocol for comparing predator effects in a similar food chain [4], and these organisms co-occur in highly contaminated estuaries like San Francisco Bay. We examined the separate and cumulative effects of predators (e.g. consumptive, non-consumptive, and total effects) on prey consumption rates, and how these changed with copper pollution.

## Material and Methods

We examined the influence of copper pollution on the cascading effects of predatory rock crabs, *Cancer productus*, on their invasive whelk prey, *Urosalpinx cinerea*, and the barnacle prey of whelks, *Balanus glandula*. These species co-occur in contaminated (e.g. San Francisco Bay [36–38]) and relatively less contaminated bays (e.g. Tomales Bay). We conducted mesocosm experiments at Bodega Marine Laboratory (Bodega Bay, CA, USA) from July—October 2013. Organisms were collected from less contaminated sites to minimize the potential for pollution tolerance of animals from more polluted sites. Crabs (carapace width range: 10–15 cm) were collected at Spud Point Marina in Bodega Harbor (38° 19' 42.80" N, 123° 3' 20.05" W). Whelks are absent from Bodega Harbor and were collected from Tomales Bay, CA (38° 7' 41.56" N, 122° 51' 49.97" W). Rocks covered with barnacles were collected at an intertidal cobble beach in Bodega Harbor (38° 18' 17.98" N, 123° 3' 26.15" W). All experiments were conducted in a 16°C cold room with a 12:12 L:D light cycle. Water was recirculated in mesocosms to minimize the production of chemical waste. This study was carried out in strict accordance with the recommendations and guidelines of the Bodega Marine Reserve and Laboratory of the University of California, Davis.

### Experiment 1: Influence of whelk conspecific density on per capita whelk consumption rates

Prior to examining the impact of copper pollution on conspecific interactions, we confirmed that whelks responded to conspecifics in the absence of copper pollution. We used mesocosms to measure the influence of conspecific density on per capita whelk consumption of barnacles. Each mesocosm consisted of two containers (32 x 15 x 12 cm) containing a total of 7L of seawater. An aquarium pump in each downstream container pumped water to the upstream container. Water drained passively from the upstream to the downstream container at ~1.5 L minute^-1^. Four densities of whelks were added to the downstream containers (1, 2, 5, or 10 whelks; n = 5). We placed a rock (~5 cm diameter) covered with barnacles (35 ± 1 barnacles per rock, mean ± SEM) as prey for whelks in each downstream container. Ten whelks were placed in upstream containers for all treatments to attempt to saturate the mesocosms with waterborne cues from conspecific whelks.

After seven days, we counted the number of barnacles consumed in each replicate. Whelk per capita consumption rates of barnacles were calculated by dividing the total number of barnacles consumed with the number of experimental whelks in each downstream container. We performed an ANOVA with whelk density treatments as the independent categorical variable and whelk per capita consumption of barnacles as the response variable. We compared consumption rates among each whelk density using Tukey HSD post hoc tests. We performed all statistical analysis with JMP version 10 (SAS Institute Incorporated, Cary, NC, USA).

### Experiment 2: Influence of copper concentration on prey responses to predation risk and crab predation rates

To examine the influence of copper pollution on 1) crab predator non-consumptive effects on whelk prey and 2) crab predation rates of whelks, we conducted a fully orthogonal mesocosm experiment that manipulated Copper (0, 10, 25, 50, or 100 ppb; n = 5) and Crab (Crab, No Crab; n = 5). Each replicate consisted of an upstream and downstream container (same set-up as above). We added five whelks and rocks (~5 cm diameter) covered with barnacles (31 ± 2, mean ± SEM) to each downstream container. We placed a single crab and 10 live, intact whelks as prey to each upstream container for Crab treatments. We added 10 whelks to the upstream container of both Crab and No Crab treatments at the beginning of the experiment. Because crabs consumed most of the whelks within two days, we added an additional 10 whelks to each upstream container of Crab treatments on Days 2, 4, and 6. This design allowed us to examine predator non-consumptive effects because downstream whelks in Crab treatments could encounter chemical cues from crab predators and attacked conspecifics without being vulnerable to crab predation. After seven days, we measured the number of barnacles consumed by whelks in the downstream containers to examine the indirect effect of crab cues on barnacles, and how this changed with copper pollution. We also counted the number of whelks consumed by crabs in upstream containers to examine the effect of copper on crab predation rates of whelks.

To manipulate Copper, we added 0, 10, 25, 50, or 100 ppb of dissolved copper made from copper (II) chloride dehydrate crystals (>99.0% purity; Sigma-Aldrich, St. Louis, MO, USA) to the seawater of each mesocosm. Thus, all three species were exposed to copper treatments simultaneously. The seawater source was Horseshoe Cove (38° 19' 00.20" N, 123° 04' 15.60" W), a site adjacent to the Bodega Marine Laboratory. Quarterly pollutant tests of seawater around the Bodega Marine Laboratory indicated total copper levels < 0.5 ppb (K. Brown, personal communication). Our range of concentrations includes the highest dissolved copper levels found in coastal environments in California (20 ppb; [39]), the United States (50 ppb; [40]), and the United Kingdom (176 ppb; [41]). Although our highest copper concentration (100 ppb) is rarely encountered in most coastal environments in the USA, such concentrations are not unknown for urban runoff into estuaries around the world [41,42].

To maximize our replication despite space limitations, we conducted the experiment twice: in July (n = 3) and August 2013 (n = 2). Because we did not find any interactive effects of any treatment (Crab or Copper) with time, we combined all five replicates for the statistical analysis. To examine the impact of pollution on predator non-consumptive effects, we performed an ANCOVA with two fixed factors: Crab (categorical variable) and Copper concentration (continuous variable). Our response variable was the total number of barnacles consumed by whelks. To better understand this interaction, we compared the relationship between Copper and total barnacles consumed at each level of Crab treatments separately using linear regression. Additionally, we used an ANOVA to test whether Copper treatments (0, 10, 25, 50, or 100 ppb) impacted crab predation rates.

### Experiment 3: Influence of copper on the relative strength of predator consumptive and non-consumptive effects

To simultaneously compare the effect of copper on the consumptive effects, non-consumptive effects, and total effects of crab predators, we conducted another fully factorial orthogonal experiment using the above mesocosm set-up. Each replicate started with five focal whelks and a barnacle-covered rock in the downstream container. Initially, experimental rocks contained 24 ± 1 barnacles (mean ± SEM). We manipulated Crab (Crab, No Crab; n = 5), Copper (0, 50 ppb; n = 5), and Culling (Cull, No Cull; n = 5). Crab and Copper treatments were manipulated as described above. For this experiment, we only used 50 ppb for our Copper treatment because the previous experiment showed evidence that this was the lowest concentration that affected these interactions. The Culling treatment allowed us to determine predator consumptive effects independent of their non-consumptive effects. For Culling treatments, we removed two whelks on Day 2 and Day 4 of the seven day experiment (average removal rates of 0.57 whelks per day). These removal rates matched crab consumption rates of whelks found in the Experiment 2. We recognize that individual whelks likely experience a smaller probability of predation on a population-wide basis. As a result, our estimates of consumptive effects are likely representative of localized areas with intense crab predation rather than representative of the population-wide consumptive effects. No whelks were removed in the No Culling treatments.

After seven days, we recorded the number of barnacles consumed by the whelks for each replicate. Then, we calculated the per capita whelk consumption rates of barnacles (R) by dividing the number of barnacles consumed with the average number of focal whelks in each replicate. These per capita consumption rates for each treatment were used to calculate the effect size of predator Consumptive (CE), Non-Consumptive (NCE), and Total Effects (TE) using the following equations (see [4,23,43] for similar metrics):

$$CE\;=\;1\;-\;\left(R_{no}{}_{crab,\;cull}/\;R_{no\;crab,\;no\;cull}\right)$$

$$NCE\;=\;1\;-\;\left(R_{crab,\;no\;cull}/\;R_{no\;crab,\;no\;cull}\right)\;TE\;=\;1\;-\;\left(R_{crab,\;cull}/\;R_{no\;crab,\;no\;cull}\right)$$

Thus, these indices determined the proportional reduction of per capita whelk consumption of barnacles caused by each predator effect type. For example, a CE = 0.40 indicates whelk removal decreasing consumption rates by 40%. Likewise, a NCE = 0.28 corresponds to crab cues reducing consumption rates by 28%. And a TE = -0.08 means the combination of whelk removal and crab cues increasing consumption rates by 8%.

We calculated the relative importance of predation risk, in either No Copper (0 ppb) or Copper (50 ppb), by dividing NCE values of each replicate with the sum of the average absolute values of NCE and CE (see [5] for similar metrics). We also counted the number of whelks consumed by crabs in upstream containers to examine the effect of copper on crab predation rates. To maximize our replication despite space limitations, we conducted the experiment twice: in September (n = 3) and October 2013 (n = 2). Because we did not find any interactive effects of any treatment (Crab, Culling, or Copper) with time, we combined all five replicates for the statistical analysis.

We performed a three-way ANOVA with Copper, Crab, and Culling as categorical fixed variables and per capita whelk consumption of barnacles as the response variable. We explored the three-way interaction by conducting two-way ANOVAs separately for the Crab and No Crab treatments (see [44] for similar metrics). When these two-way ANOVAs detected a statistically significant interaction, we followed these tests with two-sample t-tests. We conducted a two-way ANOVA on the effect size of the Predator Effect Type (CE, NCE, or TE) under Copper treatments. We treated these Predator Effect Type and Copper treatments as fixed variables. We performed Tukey HSD post hoc tests. Additionally, we used a two-way ANOVA to test whether Copper and Culling treatments impacted crab predation rates of whelks.

## Results

### Experiment 1: Influence of conspecific density on per capita whelk consumption rates

**Fig 1:**
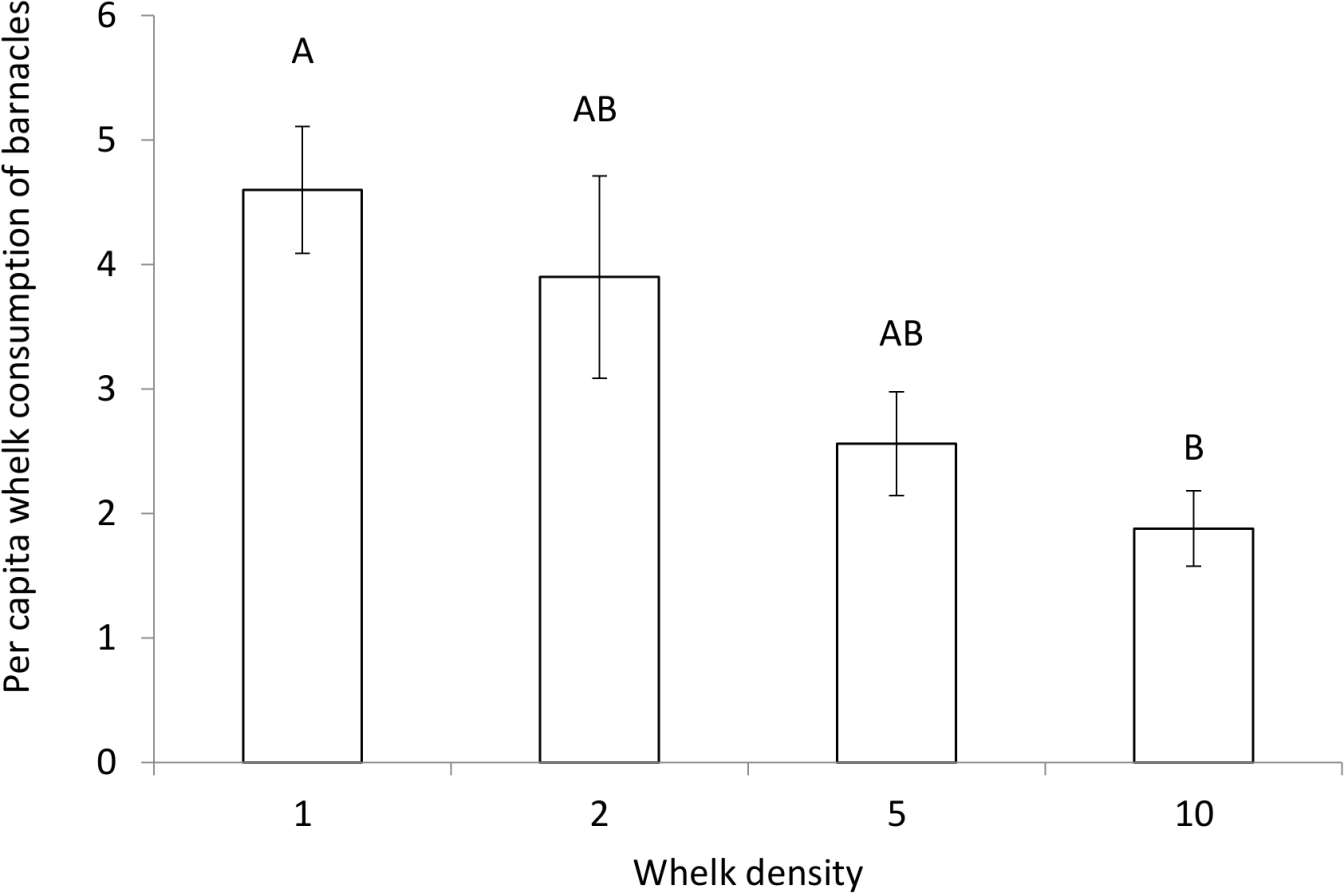
Effects of whelk conspecific density on whelk consumption rates. Mean (±SEM) per capita whelk consumption of barnacles in 1, 2, 5, and 10 whelk density treatments (n = 5) after seven days. Different letters indicate significantly different groups (Tukey HSD tests, p < 0.05).

Whelk behavior changed with conspecific density in the absence of copper. On a per capita basis, whelks consumed more barnacles with decreasing whelk density (ANOVA, F_3,16_ = 5.17, p = 0.011, Fig 1). Whelks foraging alone had twice the per capita consumption rates of whelks foraging in groups of 10 (Fig 1).

### Experiment 2: Influence of copper concentration on prey responses to predation risk and crab predation rates

**Fig 2:**
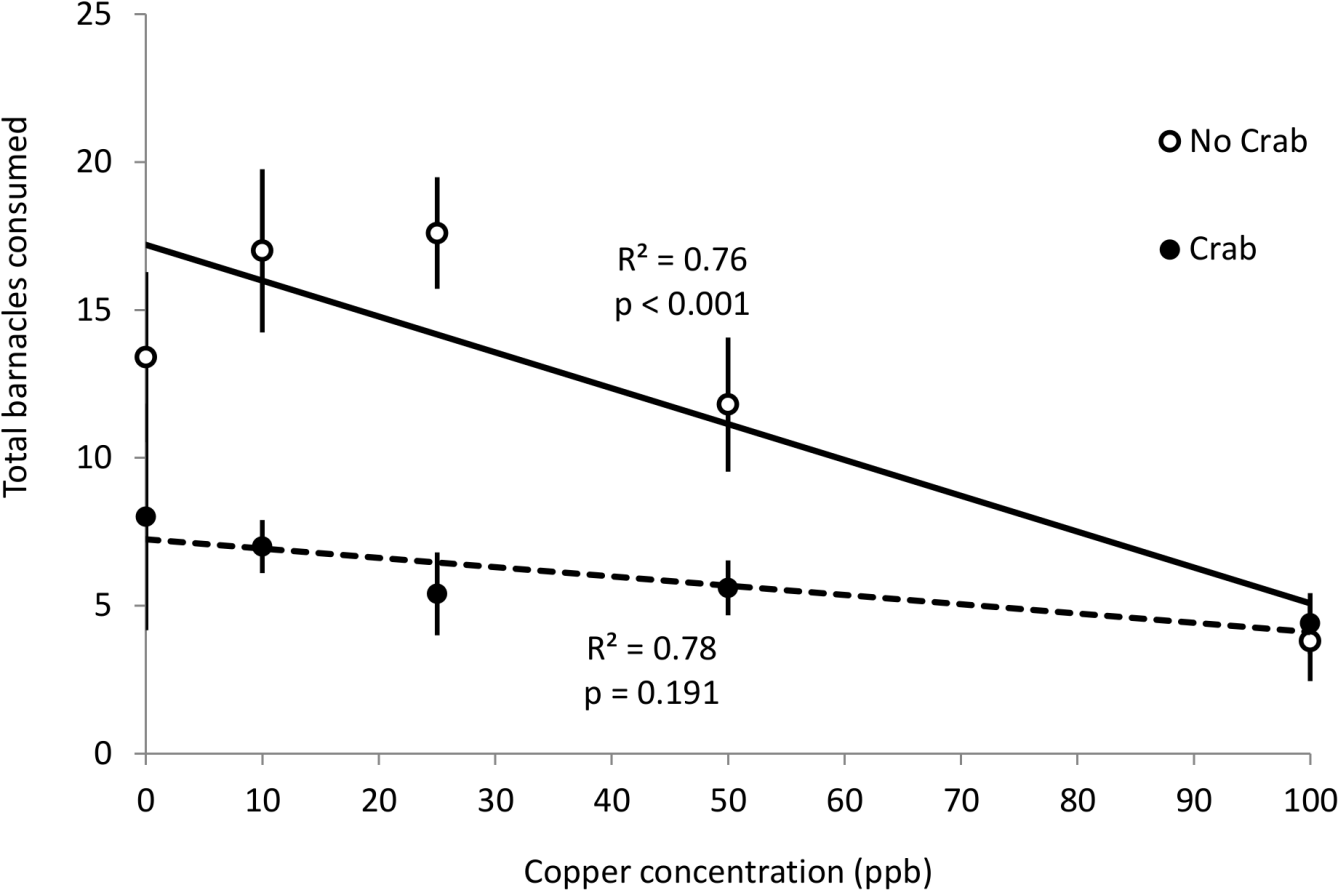
Effects of crab predator and copper concentrations on whelk consumption rates. Mean (±SEM) total number of barnacles consumed by whelks under No Crab (empty circles; n = 5) and Crab (filled circles; n = 5) treatments when exposed to five different copper concentration treatments (n = 5). R^2^ and p-values are from a linear regression of the five copper concentrations with either No Crab (solid line) or Crab (dashed line) treatments. Dashed line for Crab treatments shown for clarity although not a statistically significant regression.

Predation risk from crabs reduced whelk consumption of barnacles (Fig 2; S1 Table). However, these crab non-consumptive effects depended upon copper concentration (Crab*Copper interaction, Fig 2; S1 Table). For No Crab treatments, whelks consumed fewer barnacles with increasing copper concentration (ANOVA, F_1,23_ = 15.98, p < 0.001; linear regression, R^2^ = 0.76, p < 0.001, Fig 2). In contrast, whelks exposed to predation risk ate consistently across copper levels (ANOVA, F_1,23_ = 0.88, p = 0.36; linear regression, R^2^ = 0.78, p = 0.191, Fig 2).

Crab predation on whelks did not depend upon copper concentration (linear regression, R^2^ = 0.01, p = 0.74). Crabs consumed 16 (±4), 8 (±4), 13 (±4), 12 (±4), and 11(±5) whelks [mean (±SEM) for 0, 10, 25, 50, and 100 ppb of copper, respectively].

### Experiment 3: Influence of copper on the relative strength of predator consumptive and non-consumptive effects

**Fig 3:**
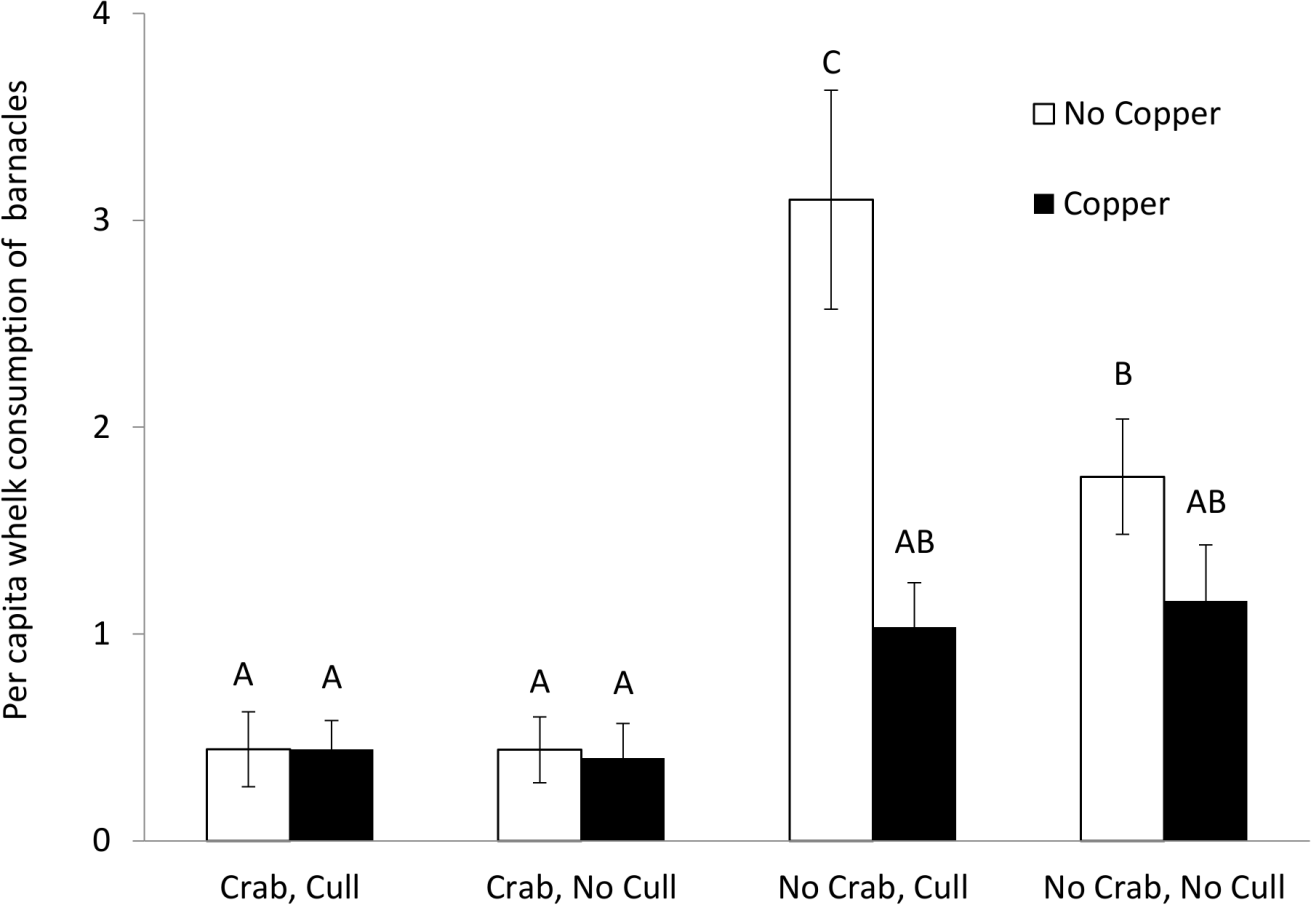
Effects of copper, crab predator, and culling on whelk consumption rates. Mean (±SEM) per capita whelk consumption of barnacles under Copper (No Copper, Copper; n = 5), Crab (Crab, No Crab; n = 5), and Culling (Cull, No Cull; n = 5) treatments. Empty bars represent No Copper treatments. Filled bars represent 50 ppb Copper treatments. Per capita whelk consumption of barnacles was calculated as total number of barnacles consumed by whelks divided with the average number of experimental whelks in each replicate. Different letters indicate significantly different groups (Tukey HSD tests, p < 0.05).

Consistent with the previous experiment, crab cues decreased per capita whelk consumption rates of barnacles (Fig 3; S2 Table). There was suggestion at α = 0.05 of a three-way interaction among Copper, Crab, and Culling treatments (three-way ANOVA, p = 0.058, Fig 3; S2 Table). When this three-way interaction was explored with separate two-way ANOVAs at each level of Crab treatments (Crab, No Crab), we observed a statistically significant interaction of Copper and Culling in the absence of crab cues (p = 0.050; S3 Table) but not in their presence (p = 0.904; S3 Table). In No Crab treatments, Culling increased consumption rates in the absence (two-sample t-test, t_5.29_ = 3.61, p = 0.014) but not the presence of Copper (two-sample t-test, t_7.99_ = 1.54, p = 0.161).

**Fig 4:**
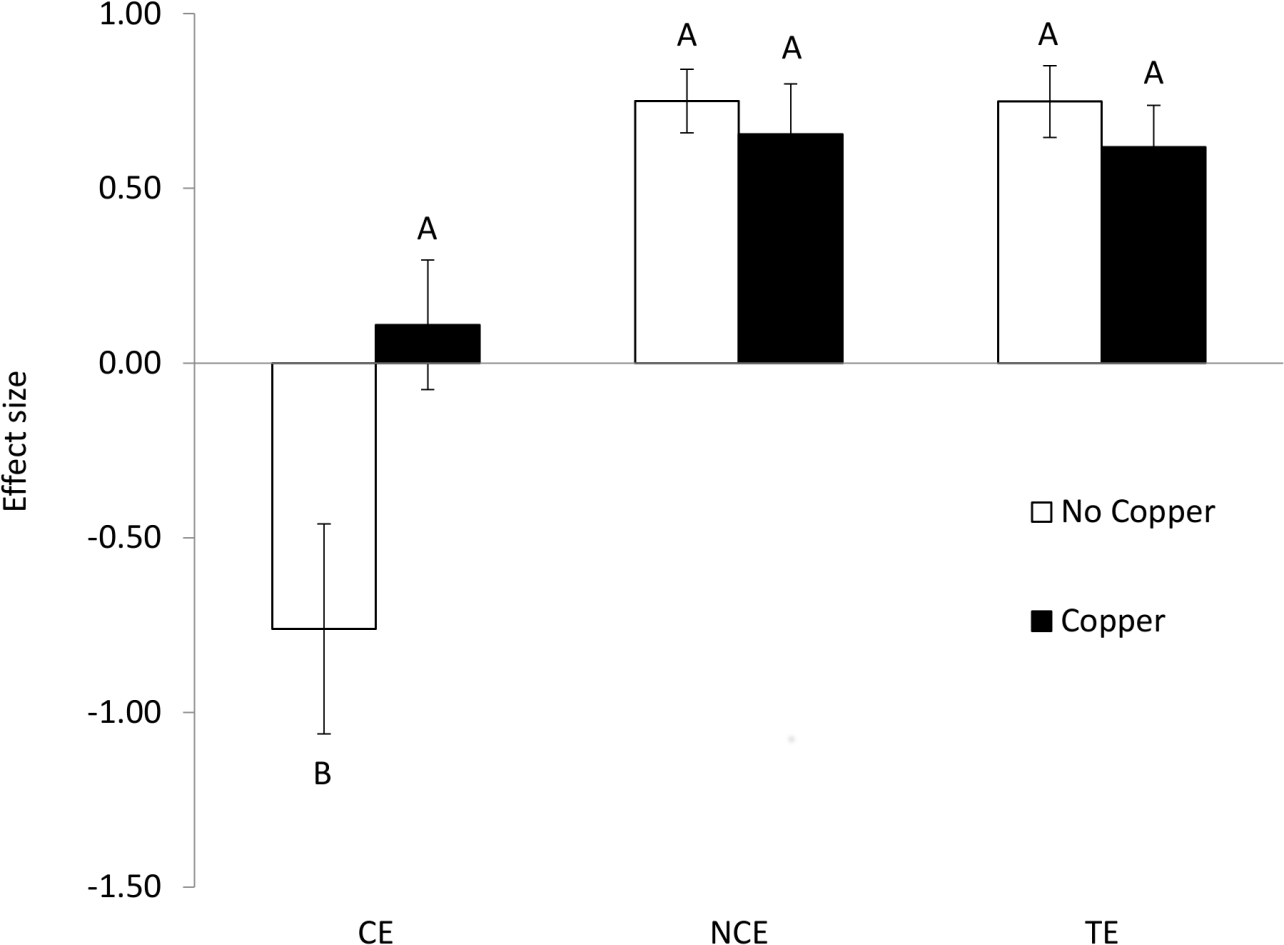
Effects of copper and crab predator effect type on whelk consumption rates. Mean (±SEM) effect size of crab predator Consumptive Effects (whelk culling, CE; n = 5), Non-Consumptive Effects (crab cues, NCE; n = 5), and Total Effects (crabs cues and whelk culling, TE; n = 5) calculated with per capita whelk consumption of barnacles. Empty bars represent No Copper treatments (n = 5). Filled bars represent 50 ppb Copper treatments (n = 5). Different letters indicate significantly different groups (Tukey HSD tests, p < 0.05).

Copper and Predator Effect Type (Consumptive, Non-Consumptive, or Total Effects) interacted to influence the magnitude of the effects of predators on per capita whelk consumption of barnacles (F_2,24_ = 5.44, p = 0.011, Fig 4; S4 Table). Adding copper removed the negative Consumptive Effects of predators, because whelks did not increase per capita consumption when culling decreased conspecific density (Fig 4). In contrast, copper had no effect on the magnitude of the Non-Consumptive and Total Effects of predators (Fig 4). The relative importance of predation risk was greater in Copper than No Copper treatments (0.87 ± 0.19 and 0.50 ± 0.06, respectively; mean ± SEM)–though this comparison was marginally significant at α = 0.05 (two sample t-test, t_4.80_ = -1.86, p = 0.062).

Copper, Culling, and the Copper*Culling interaction did not significantly affect crab predation rates of whelks (Copper*Culling interaction, two-way ANOVA, F_1,16_ = 0.12, p = 0.74). Copper did not affect crab predation rates (F_1,16_ = 0.00, p = 1.00). Neither did Culling affect crab predation rates (F_1,16_ = 1.12, p = 0.31). Crabs consumed 24.3 (±4.3) whelks in Copper treatments and 24.3 (±2.9) whelks in No Copper treatments [mean (±SEM)].

## Discussion

Copper pollution altered the relative strengths of crab predator consumptive and non-consumptive effects. Because sublethal, intermediate copper levels did not alter the effects of predation risk or crab predation of whelks, this shift originated from copper changing the effects of simulated lethal crab predation (culling) on whelks. In the absence of copper, these predator consumptive effects on prey consumption rates were large and negative because prey increased their foraging rate with decreasing conspecific density. Surprisingly, this strong predator consumptive effect was removed completely with the addition of copper. Thus, copper pollution at 50 ppb decreased the relative role of lethal predation and increased the relative importance of predation risk. These results appear to contrast with findings that non-consumptive effects weakened during exposure to copper [12,13], pesticides [45], and ocean acidification [46]. However, our experiments did find a reduction in non-consumptive effects at extremely high copper levels (100 ppb), suggesting that predation risk may be more sensitive to chemical contamination for fishes [12,13] than whelks (this study). Together, our findings suggest that the ways in which predators can affect communities may be differentially influenced by copper pollution, namely, predator consumptive effects were more sensitive to copper pollution.

Because energy transfer and nutrient cycling depend upon how predators affect prey (i.e. eating them versus scaring them; [1,5,6]), stressor-mediated shifts in the relative role of predation risk could alter community structure and function. These outcomes will also be determined by the direct impact of stressors on organisms. In our study, copper had strong effects on the behavior of whelks but no lethal effects on any of the three species tested. Copper also did not alter crab predation rates on whelks. Similar to our findings, a recent study found that increased temperatures associated with climate change largely impacted marine communities through changes in the behavior and performance of species, rather than via changes in survivorship [7].

Our results also suggest that extreme copper concentrations (>50 ppb) may dampen the consequences of fear release caused by predator removal from aquatic ecosystems. Fear release occurs when prey alter their behavior and distribution as predators are removed from communities. For example, shark declines are predicted to shift the preferred foraging strata of seals deeper as they seek more predictable fish resources [47]. Similarly, fear release from crabs (e.g. due to overfishing) in our system should lead to an increase in per capita whelk consumption of barnacles (Figs 2 and 3). However, this increase failed to occur under our highest copper concentrations (Fig 2), suggesting that predator removal and copper pollution may act antagonistically to shape prey foraging.

Consistent with other studies of marine invertebrates [5,23] we observed an inverse relationship between conspecific density and per capita consumption rates in non-polluted scenarios. This pattern may have at least two explanations. First, direct interactions (either antagonistic or beneficial) may increase with conspecific density [48], thereby decreasing feeding opportunities. Second, conspecific signaling at higher densities may have lowered per capita consumption. This latter hypothesis may be likely given that *Urosalpinx cinerea* whelks avoid areas containing conspecific chemical cues [49,50]. Regardless of the mechanism, however, our results suggest that copper removed the density-dependent behaviors of whelks.

Copper effects in our system were species-specific, with whelk prey more affected than crab predators. The source of this species-specificity is unclear but might arise because of taxonomic differences in copper sensitivity [51–53]. Our results are consistent with the finding that crabs are ~10x more tolerant to copper than gastropod molluscs [53]. At least three mechanisms may account for this taxonomic difference. First, arthropods and molluscs may process pollutants differently. Crabs may quickly excrete contaminants whereas whelks may detoxify or store pollutants [52]. Second, production of metallothionein to decrease bioavailability of copper may be more easily induced in crabs than molluscs [53]. Third, copper increases crustacean heart rates but decreases molluscan heart rates [53]. Although taxonomic differences in copper sensitivity may account for this species-specificity, we should note that we did not examine the effects of copper on long distance olfactory foraging in crabs. Given that such behaviors can critically shape predator-prey interactions [54,55], we need studies that simultaneously examine consumer-prey interactions across long and short distances [35].

### Conclusion

Despite widespread recognition that chemical pollutants increasingly threaten coastal ecosystems, we lack a basic understanding of how these anthropogenic stressors influence community dynamics. This paucity likely persists because of divergent approaches by ecologists and toxicologists. Our study helps bridge that gap between ecology and toxicology by employing a more interdisciplinary approach for a more encompassing assessment of anthropogenic impacts on ecosystems.

In contrast to our original predictions, exposing an estuarine food chain to sublethal, intermediate levels of copper increased, rather than decreased, the importance of predation risk relative to lethal predation on species interactions. This effect occurred because intermediate copper levels removed density-dependent interactions among conspecific prey but did not influence predation risk. Our findings highlight a novel mechanism by which contaminants may commonly influence the structure and function of aquatic communities, particularly given the prevalence of conspecific interactions amongst intermediate trophic levels. Our study is the first to observe chemical pollutants influence the relative strengths of predator consumptive and non-consumptive effects on prey. These findings suggest pollutants may impact ecosystems through mechanisms rarely studied by ecologists and toxicologists. Additional studies of the influence of sublethal levels of pollution on species interactions are needed to more completely assess ecosystem responses to human-caused stressors.

## Supporting Information

S1 Table ANCOVA statistics of crab and copper effects on whelk consumption rates. Results of ANCOVA testing the effect of crab chemical cues and copper concentration (covariate) on whelk consumption of barnacles in *Experiment 2*: *Influence of copper concentration on prey responses to predation risk and crab predation rates*.
(PDF)

S2 Table ANOVA statistics of copper, crab, and culling on whelk consumption rates. Results of three-way ANOVA testing the effect of copper exposure, crab chemical cues, and whelk culling treatments on per capita whelk consumption of barnacles in *Experiment 3*: *Influence of copper on the relative strength of predator consumptive and non-consumptive effects*.
(PDF)

S3 Table ANOVA statistics of copper and culling effects on whelk consumption rates for crab and no crab treatments. Results of two-way ANOVA testing the effect of copper exposure and whelk culling treatments on per capita whelk consumption of barnacles in *Experiment 3*: *Influence of copper on the relative strength of predator consumptive and non-consumptive effects*. Two-way ANOVAs were conducted for Crab and No Crab treatments separately.
(PDF)

S4 Table ANOVA statistics of the effects of copper and crab predator effect type on whelk consumptive rates. Results of two-way ANOVA testing the influence of copper exposure on the effect sizes of predator effect type (Consumptive, Non-Consumptive, or Total Effects) on per capita whelk consumption of barnacles in *Experiment 3*: *Influence of copper on the relative strength of predator consumptive and non-consumptive effects*.
(PDF)
