# Correction: Copper Pollution Increases the Relative Importance of Predation Risk in an Aquatic Food Web

**DOI:** 10.1371/journal.pone.0136006

**Published:** 2015-08-13

**Authors:** The PLOS ONE Staff

In the subsection “Experiment 3: Influence of copper on the relative strength of predator consumptive and non-consumptive effects” within the Material and Methods, the equations are incorrect. The publisher apologizes for the error. Please view the complete, correct equations here:

$$CE=1-\left(R_{no\;crab,\;cull}/\;R_{no\;crab,\;no\;cull}\right)$$

$$NCE=1-\left(R_{crab,\;no\;cull}/\;R_{no\;crab,\;no\;cull}\right)$$

$$TE=1-\left(R_{crab,\;cull}/\;R_{no\;crab,\;no\;cull}\right)$$
