# Supplementary material for: Copper Pollution Increases the Relative Importance of Predation Risk in an Aquatic Food Web
Source: PLoS One. 2015 Jul 14;10(7):e0133329. doi: 10.1371/journal.pone.0133329 (PMC4501717; doi:10.1371/journal.pone.0133329)
Supplement: S1 Table — Results of ANCOVA testing the effect of crab chemical cues and copper concentration (covariate) on whelk consumption of barnacles in Experiment 2: Influence of copper concentration on prey responses to predation risk and crab predation rates. (PDF) [file pone.0133329.s001.pdf]

S1 Table. ANCOVA statistics of crab and copper effects on whelk consumption rates.

Source	df	MS	F	p
Crab	1	551.12	23.67	<0.001
Copper	1	370.98	15.93	<0.001
Crab X Copper	1	128.84	5.53	0.023
Error	46	23.28		
