# Supplementary material for: Copper Pollution Increases the Relative Importance of Predation Risk in an Aquatic Food Web
Source: PLoS One. 2015 Jul 14;10(7):e0133329. doi: 10.1371/journal.pone.0133329 (PMC4501717; doi:10.1371/journal.pone.0133329)
Supplement: S2 Table — Results of three-way ANOVA testing the effect of copper exposure, crab chemical cues, and whelk culling treatments on per capita whelk consumption of barnacles in Experiment 3: Influence of copper on the relative strength of predator consumptive and non-consumptive effects. (PDF) [file pone.0133329.s002.pdf]

S2 Table. ANOVA statistics of copper, crab, and culling on whelk consumption rates.

Source	df	MS	F	p
Copper	1	4.57	12.52	0.001
Crab	1	17.74	48.60	<0.001
Culling	1	1.00	2.74	0.108
Copper X Crab	1	4.30	11.79	0.002
Crab X Cull	1	0.85	2.33	0.136
Copper X Cull	1	1.27	3.47	0.072
Crab X Copper X Cull	1	1.41	3.87	0.058
Error	32	0.37		
