# Supplementary material for: Copper Pollution Increases the Relative Importance of Predation Risk in an Aquatic Food Web
Source: PLoS One. 2015 Jul 14;10(7):e0133329. doi: 10.1371/journal.pone.0133329 (PMC4501717; doi:10.1371/journal.pone.0133329)
Supplement: S3 Table — Results of two-way ANOVA testing the effect of copper exposure and whelk culling treatments on per capita whelk consumption of barnacles in Experiment 3: Influence of copper on the relative strength of predator consumptive and non-consumptive effects. Two-way ANOVAs were conducted for Crab and No Crab treatments separately. (PDF) [file pone.0133329.s003.pdf]

S3 Table. ANOVA statistics of copper and culling effects on whelk consumption rates for crab and no crab treatments.

Crab Treatments

Source	df	MS	F	p
Copper	1	0.002	0.015	0.904
Culling	1	0.003	0.022	0.884
Copper X Culling	1	0.002	0.015	0.904
Error	16	0.132		

No Crab Treatments

Source	df	MS	F	p
Copper	1	8.871	14.831	0.001
Culling	1	1.848	3.090	0.098
Copper X Culling	1	2.679	4.479	0.050
Error	16	0.598		
