# Supplementary material for: Copper Pollution Increases the Relative Importance of Predation Risk in an Aquatic Food Web
Source: PLoS One. 2015 Jul 14;10(7):e0133329. doi: 10.1371/journal.pone.0133329 (PMC4501717; doi:10.1371/journal.pone.0133329)
Supplement: S4 Table — Results of two-way ANOVA testing the influence of copper exposure on the effect sizes of predator effect type (Consumptive, Non-Consumptive, or Total Effects) on per capita whelk consumption of barnacles in Experiment 3: Influence of copper on the relative strength of predator consumptive and non-consumptive effects. (PDF) [file pone.0133329.s004.pdf]

**S4 Table. ANOVA statistics of the effects of copper and crab predator effect type on whelk consumptive rates.**

| Source | df | MS | F | p |
|---|---|---|---|---|
| Copper | 1 | 0.35 | 2.34 | 0.140 |
| Predator Effect Type | 2 | 3.50 | 23.37 | <0.001 |
| Copper X Effect Type | 2 | 0.81 | 5.44 | 0.011 |
| Error | 24 | 0.15 | | |
